# Supplementary material for: HIVprotI: an integrated web based platform for prediction and design of HIV proteins inhibitors
Source: J Cheminform. 2018 Mar 9;10:12. doi: 10.1186/s13321-018-0266-y (PMC5845081; doi:10.1186/s13321-018-0266-y)
Supplement: Supplementary file 2 — Additional file 2. Source code of HIVProtI web server. [file 13321_2018_266_MOESM2_ESM.zip › HIVprotI_Source-code/hivprotI/weka-3-6-6/doc/weka/gui/sql/ResultSetTableModel.html]

ResultSetTableModel


---


|  |  |  |
| --- | --- | --- |
| **Overview** \| **Package** \| **Class** \| **Tree** \| **Deprecated** \| **Index** \| **Help** \| **Weka's home** | |  |
| **PREV CLASS**   **NEXT CLASS** | **FRAMES**    **NO FRAMES**     **All Classes** |  |
| SUMMARY: NESTED \| FIELD \| CONSTR \| METHOD | DETAIL: FIELD \| CONSTR \| METHOD |  |


---


## weka.gui.sql Class ResultSetTableModel

```
java.lang.Object
  weka.gui.sql.ResultSetTableModel
```

**All Implemented Interfaces:**: javax.swing.table.TableModel

---

``` public class ResultSetTableModel extends java.lang.Object implements javax.swing.table.TableModel ```

The model for an SQL ResultSet.

**Version:**
:   $Revision: 7059 $

**Author:**
:   FracPete (fracpete at waikato dot ac dot nz)

---

**Constructor Summary**

`ResultSetTableModel(java.sql.ResultSet rs)`
  
          initializes the model, retrieves all rows.

`ResultSetTableModel(java.sql.ResultSet rs,
int rows)`
  
          initializes the model, retrieves only the given amount of rows (0 means
all).


| **Method Summary** | |
| --- | --- |
| `void` | `addTableModelListener(javax.swing.event.TableModelListener l)`             adds a listener to the list that is notified each time a change to data model occurs. |
| `void` | `finalize()`             frees up the memory. |
| `java.lang.Class` | `getColumnClass(int columnIndex)`             returns the most specific superclass for all the cell values in the column (always String). |
| `int` | `getColumnCount()`             returns the number of columns in the model. |
| `java.lang.String` | `getColumnName(int columnIndex)`             returns the name of the column at columnIndex. |
| `int` | `getRowCount()`             returns the number of rows in the model. |
| `java.lang.Object` | `getValueAt(int rowIndex, int columnIndex)`             returns the value for the cell at columnindex and rowIndex. |
| `boolean` | `isCellEditable(int rowIndex, int columnIndex)`             returns true if the cell at rowindex and columnindexis editable. |
| `boolean` | `isNullAt(int rowIndex, int columnIndex)`             checks whether the value of the cell is NULL. |
| `boolean` | `isNumericAt(int columnIndex)`             returns whether the column at the given index is numeric. |
| `void` | `removeTableModelListener(javax.swing.event.TableModelListener l)`             removes a listener from the list that is notified each time a change to the data model occurs. |
| `void` | `setValueAt(java.lang.Object aValue, int rowIndex, int columnIndex)`             sets the value in the cell at columnIndex and rowIndex to aValue. |

**Methods inherited from class java.lang.Object**

`equals, getClass, hashCode, notify, notifyAll, toString, wait, wait, wait`

**Constructor Detail**

### ResultSetTableModel

```
public ResultSetTableModel(java.sql.ResultSet rs)
```

:   initializes the model, retrieves all rows.

    **Parameters:**: `rs` - the ResultSet to get the data from

---


### ResultSetTableModel

```
public ResultSetTableModel(java.sql.ResultSet rs,
                           int rows)
```

:   initializes the model, retrieves only the given amount of rows (0 means
    all).

    **Parameters:**: `rs` - the ResultSet to get the data from: `rows` - the maximum number of rows to retrieve, 0 retrieves all


**Method Detail**

### addTableModelListener

```
public void addTableModelListener(javax.swing.event.TableModelListener l)
```

:   adds a listener to the list that is notified each time a change to data
    model occurs.

    :   **Specified by:**: `addTableModelListener` in interface `javax.swing.table.TableModel`
    :   **Parameters:**: `l` - the listener to add

---


### getColumnClass

```
public java.lang.Class getColumnClass(int columnIndex)
```

:   returns the most specific superclass for all the cell values in the
    column (always String).

    :   **Specified by:**: `getColumnClass` in interface `javax.swing.table.TableModel`
    :   **Parameters:**: `columnIndex` - the index of the column **Returns:**: the class

---


### getColumnCount

```
public int getColumnCount()
```

:   returns the number of columns in the model.

    :   **Specified by:**: `getColumnCount` in interface `javax.swing.table.TableModel`
    :   **Returns:**: the number of columns

---


### getColumnName

```
public java.lang.String getColumnName(int columnIndex)
```

:   returns the name of the column at columnIndex.

    :   **Specified by:**: `getColumnName` in interface `javax.swing.table.TableModel`
    :   **Parameters:**: `columnIndex` - the index of the column **Returns:**: the name

---


### getRowCount

```
public int getRowCount()
```

:   returns the number of rows in the model.

    :   **Specified by:**: `getRowCount` in interface `javax.swing.table.TableModel`
    :   **Returns:**: the number of data rows

---


### getValueAt

```
public java.lang.Object getValueAt(int rowIndex,
                                   int columnIndex)
```

:   returns the value for the cell at columnindex and rowIndex.

    :   **Specified by:**: `getValueAt` in interface `javax.swing.table.TableModel`
    :   **Parameters:**: `rowIndex` - the row of the cell: `columnIndex` - the column of the cell **Returns:**: the data value

---


### isNullAt

```
public boolean isNullAt(int rowIndex,
                        int columnIndex)
```

:   checks whether the value of the cell is NULL.

    :   **Parameters:**: `rowIndex` - the row of the cell: `columnIndex` - the column of the cell **Returns:**: true if the cell value is NULL

---


### isNumericAt

```
public boolean isNumericAt(int columnIndex)
```

:   returns whether the column at the given index is numeric.

    :   **Parameters:**: `columnIndex` - the column to check **Returns:**: whether the column is numeric

---


### isCellEditable

```
public boolean isCellEditable(int rowIndex,
                              int columnIndex)
```

:   returns true if the cell at rowindex and columnindexis editable.

    :   **Specified by:**: `isCellEditable` in interface `javax.swing.table.TableModel`
    :   **Parameters:**: `rowIndex` - the row of the cell: `columnIndex` - the column of the cell **Returns:**: always false

---


### removeTableModelListener

```
public void removeTableModelListener(javax.swing.event.TableModelListener l)
```

:   removes a listener from the list that is notified each time a change to
    the data model occurs.

    :   **Specified by:**: `removeTableModelListener` in interface `javax.swing.table.TableModel`
    :   **Parameters:**: `l` - the listener to remove

---


### setValueAt

```
public void setValueAt(java.lang.Object aValue,
                       int rowIndex,
                       int columnIndex)
```

:   sets the value in the cell at columnIndex and rowIndex to aValue.
    Ignored.

    :   **Specified by:**: `setValueAt` in interface `javax.swing.table.TableModel`
    :   **Parameters:**: `aValue` - the value to set - ignored: `rowIndex` - the row of the cell: `columnIndex` - the column of the cell

---


### finalize

```
public void finalize()
              throws java.lang.Throwable
```

:   frees up the memory.

    :   **Overrides:**: `finalize` in class `java.lang.Object`
    :   **Throws:**: `java.lang.Throwable` - if something goes wrong


---


|  |  |  |
| --- | --- | --- |
| **Overview** \| **Package** \| **Class** \| **Tree** \| **Deprecated** \| **Index** \| **Help** \| **Weka's home** | |  |
| **PREV CLASS**   **NEXT CLASS** | **FRAMES**    **NO FRAMES**     **All Classes** |  |
| SUMMARY: NESTED \| FIELD \| CONSTR \| METHOD | DETAIL: FIELD \| CONSTR \| METHOD |  |


---
